# Use of Levodopa After a Stroke: A Systematic Review

**DOI:** 10.7759/cureus.24529

**Published:** 2022-04-27

**Authors:** Juan A Moncayo, Mario Yepez, Mikaela Camacho, Alex S Aguirre, Diego Ojeda, Juan Fernando Ortiz, Meghdeep Sen, Jennifer Argudo, Lucia Proano, Steven Cordova, Nishel Kothari

**Affiliations:** 1 Neurology, Pontificia Universidad Católica del Ecuador, Quito, ECU; 2 Faculty of Health Sciences, Universidad Católica Santiago de Guayaquil, Guayaquil, ECU; 3 Neurology, Universidad San Francisco De Quito, Quito, ECU; 4 Medical School, Universidad San Francisco de Quito, Quito, ECU; 5 Neurology, Universidad San Fracisco de Quito, Quito, ECU; 6 Neurology, Universidad San Francisco de Quito, Quito, ECU; 7 Neurology, Larkin Community Hospital, Miami, USA; 8 Medicine, American University of Antigua, St. John's, ATG; 9 Child Neurology, Universidad de Cuenca, Cuenca, ECU; 10 Internal Medicine, Pontificia Universidad Catolica del Ecuador, Queens, USA; 11 Neurolgoy, Jawaharlal Nehru Medical College, Belgaum, IND

**Keywords:** stimulant, wakefullness, recovery, levodopa, stroke

## Abstract

Stroke is a leading cause of death and disability, especially in certain ethnic groups. Impaired consciousness is a common outcome in stroke patients, serving as a predictor of prognosis and mortality. Lately, there has been increased interest in drugs such as Levodopa (LD), which have been found to promote wakefulness. To further appreciate this association, we gathered updated evidence of this novel therapeutic approach and compared it, evaluating its clinical use in an acute stroke setting. We carried out a systematic review of clinical trials conducted exclusively on stroke patients who received levodopa. Four clinical trials were reviewed and analyzed after applying the inclusion/exclusion criteria. The use of levodopa showed positive results in four of the clinical trials, and statistically significant results in 3/4 of the studies; however, more studies need to be conducted to corroborate these results.

## Introduction and background

This article is a descriptive, systematic review of the use of wakefulness-promoting agents in the post-stroke setting. Several studies done in animal models are listed below where they have shown promising results. As stroke is the fifth cause of death in the United States with ischemic stroke representing 85% [1], this topic has converged into our scope as it should be more studied. Nevertheless, in the last 35 years, stroke mortality and incidence have decreased substantially. Among patients with Medicare coverage over 65 years, it has been reduced by 40% from 1988 to 2008 [2].

Impaired consciousness is a common outcome in stroke patients. Consciousness is the capacity to relate to one’s environment and oneself, mainly divided into two: awareness (environment) and arousal (alertness) [3]. Awareness and arousal are dependent on the integrity of the ascending reticular activating system, the medulla, and the thalamic pathways [4]. A problem in any of these paths can lead to arousal disorders [4]. The clinical analysis of these alterations can help determine the location of the infarctions, for example, severe impairment of consciousness guides us to a large hemispheric or bilateral brain stem lesion [5,6].

Levodopa (LD) is synthetized from the amino acid L-tyrosine [7]. LD is a synthetic amino acid that promotes the synthesis of dopamine, norepinephrine, and epinephrine. Dopamine is then produced by the decarboxylation of LD by aromatic L-amino acid decarboxylase in the presynaptic terminal of the substantia nigra. It is released and binds to the dopamine receptors in the postsynaptic terminal of the striatum [7]. LD was first used in animal models by the Nobel Prize winner Arvid Carlsson in 1950. In 1969, George Corzias introduced its use in humans, specifically in individuals with Parkinson’s Disease [8,9].

We conducted this systematic review to investigate the use of LD in patients with stroke, reviewing updated evidence of this novel therapeutic approach and its clinical use.

## Review

Materials and methods

Protocol

We carried out a systematic review using the PRISMA protocol.

Eligibility Criteria and Study Selection

We used clinical trials conducted on humans and written in English. Animal studies were excluded, as well as articles that did not fulfill the aims of our study. Then we only included articles about stroke patients who had been treated with LD. Assessment of motor and mood outcomes; secondary outcomes: functional, hospital stay, fatigue.

*Database and Search Strategy*

We used the PubMed database for this systematic and meta-analysis review. The search was conducted between March 1 and March 15, 2022. We used an advanced search strategy with the following terms: ("stroke"[Title/Abstract] AND "levodopa carbidopa"[Title/Abstract]) OR ("stroke"[Title/Abstract] AND "levodopa"[Title/Abstract]) OR ("ischemic stroke"[Title/Abstract] AND "levodopa"[Title/Abstract]).

*Data Extraction and Analysis*

We collected the following information from each paper: the author/year, methods, number of participants, and study design. We also extracted the main results, including the outcome measures and main limitations in each observational/clinical trial. We analyzed the studies' primary and secondary goals and gathered the main conclusions from each study.

Bias Assessment

In clinical trials, we used the Cochrane Collaboration risk-of-bias tool to assess the bias encountered, and in observational studies, we used Robins-1.

Results

**Figure 1:**
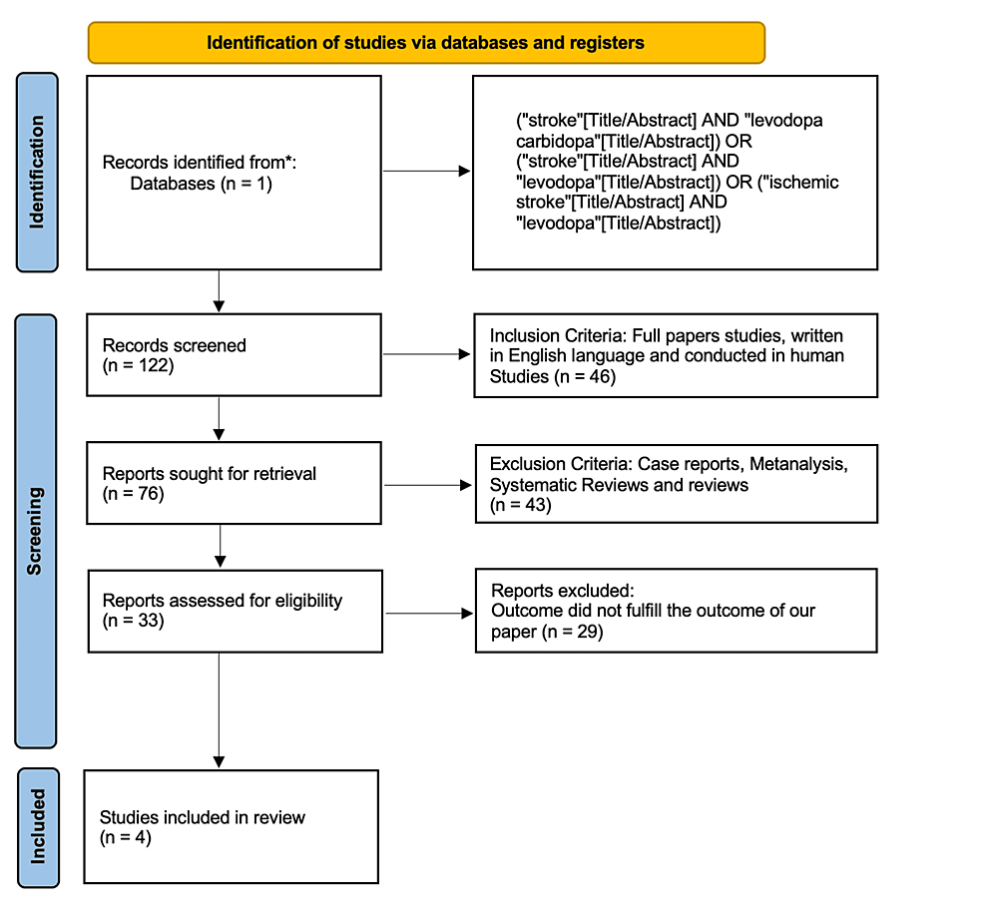
PRISMA flow chart of the systematic review

Figure 1 shows the results of the study using a PRISMA flow chart.

Study Characteristics

We found four clinical trials that specifically discussed the role of LD in the acute setting of ischemic stroke.

Table 1 presents the main characteristics of each study.

**Table 1:** Clinical trials for the use of Levodopa for stroke

Reference and country	Study design	Number patients	Number of controls
Scheidtmann et al. [10], Germany	Double blind clinical trial	22	25
Delbari et al. [11], Iran	Double blind clinical trial	78	19
Lokk et al. [12], Iran	Double blind clinical trial	78	19
Sonde and Lökk [13], Sweden	Double blind clinical trial	30	7

Table 2 shows the outcomes and conclusions of the clinical trials included in this systematic review.

**Table 2:** Outcomes of the clinical trials of Levodopa in stroke LD: Levodopa, MPD: methylphenidate, BI: Barthel index, MMSE: mini-mental state examination, GDS: geriatric depression scale, NIHSS: National Institutes of Health Stroke Scale, FM: Fugl-Meyer.

Author, Year, Country	Methods	Treatment	Outcome
Scheidtmann et al. [10], Germany	Prospective, randomized, placebo-controlled. Motor recovery was evaluated with Rivermead motor assessment.	First three weeks: LD 100 mg or placebo + physiotherapy. Second three weeks: physiotherapy only.	At three weeks, motor recovery improved by 6.4 points (p<0.004). At six weeks the motor recovery was 8.2 points (p=0.02).
Delbari et al. [11], Iran	Prospective, four armed, randomized, placebo-controlled. Mood was assessed by the GDS. Cognitive function was assessed by means of the MMSE.	Patients were divided into four groups: (a) MPD, (b) LD, (c) MPD plus LD, (d) placebo + physiotherapy for a total of 15 days (five days a week) for a course of three weeks.	Cognitive status improved in all four groups, with no significant differences between groups (p>0.1). Mood improved in the MPD plus LD group at 90 days (p<0.018) and at 180 days (p<0.006).
Lokk et al. [12], Iran	Prospective, randomized, four-armed, placebo-controlled. Activities of daily living was assessed by the BI. Stroke severity was evaluated by the NIHSS.	Patients were divided into four groups: (a) MPD, (b) LD, (c) MPD plus LD, (d) placebo plus, physiotherapy for a total of 15 days (five days a week) for a course of three weeks.	BI and NIHSS scores improved significantly at six months (p=0.011 and p=0.001, respectively) compared to baseline in the ‘Methylphenidate plus Levodopa’ group.
Sonde and Lökk [13], Sweden	Prospective, randomized, four armed, placebo controlled. Motor function was assessed with the FM scale. Activities of daily living (autonomy) was assessed by the Barthel Index. Cognition was evaluated by the NIHSS.	Patients were divided into four groups: (a) amphetamine; (b) amphetamine plus levodopa; (c) Levodopa; (d) Placebo. Patients received drugs or placebo five times a week (every working day) for two weeks and 30 min of physiotherapy one hour after intake.	No significant changes were found.

Two studies showed a clinical improvement with the use of levodopa. Scheidtmann et al. demonstrated increased motor recovery while Lokk et al. showed improvement in the functional outcome (BI and NIHSS) with the combination of LD + MPD [10,12]. Other studies did not find clinical significance regarding the functional outcome or cognitive status [11,13].

Discussion

LD is the precursor of dopamine and it is frequently used by clinicians to treat Parkinson’s disease. Once converted to dopamine, it binds to the postsynaptic dopaminergic receptors in the basal ganglia, exerting numerous effects, including wakefulness, neuroplasticity, reward system, motor, and mood modulations [14-16].

Scheidtmann et al. found that after using a single dose of LD and physiotherapy, there was an improvement in motor recovery [10]. Several hypotheses have been proposed to explain this mechanism: (i) dopamine modulates neuroplasticity as it is exemplified by the triple stimulus of glutamate at the striatal dendrite spine, postsynaptic depolarization, and dopamine release causes the spine to grow [14], and Scheidtmann et al. preferred to use a single dose of LD to avoid a steady-state concentration and proposed the idea; (ii) stimulation of norepinephrine receptors down-regulates the noradrenergic system, thus decreasing neuroplasticity; (iii) another hypothesis analyzes the prediction error system, which responds to dopaminergic bursts to induce learning [15]. However, this study did not account for specific stroke subtypes and how they can affect motor recovery. Side effects were assessed on daily ward rounds as all the subjects were inpatients.

The clinical trial by Lokk et al., which also assessed motor recovery with a larger sample size than Scheidtmann et al. [10], found that the combination of LD and MPD was statistically significantly greater than the use of LD alone. MPD acts as a norepinephrine/dopamine reuptake inhibitor, supporting the hypothesis, and possibly potentiating the neuroplasticity effects of these amphetamines [12]. The clinical trial by Sonde and Lökk was designed similarly, with a population of 36 patients. They found no benefit on the Fugl-Meyer motor scale or Barthel Index scale, although the smaller population size could explain the lack of positive results [12]. In the trials done by Lokk et al. and Delbari et al., which used the same database, all potential adverse reactions were monitored, assessed, and recorded, without any significant major side effects of these medications [11,12].

The study by Delbari et al. found that the combination of LD and MPD significantly improved mood scores on the Geriatric Depression scale. The rapid response in these patients could make it a great alternative to classic selective serotonin reuptake inhibitors (SSRIs), which might take up to six weeks to induce mood changes [11]. The author attributes the lack of effect of cognitive improvement to the decreased sensitivity of the MMSE due to its lack of evaluation of executive functions, including abstract thinking, problem-solving, judgment, and perception. Functional and mood recovery might be explained by the following mechanisms: (a) promotion of neural plasticity, (b) decreased utilization of glucose, and (c) increased mood and motivation [10,12].

Animal studies also support the idea that LD promotes neuroplasticity. Talhada et al. used Wistar rats as subjects, causing transient occlusion of the middle cerebral artery. The investigators assessed the Nogo-A pathway in these subjects. The Nogo-A pathway downregulates neuronal outgrowth, dendritic spine formation, and the oligodendrocyte transcription factor [17]. The study found that the use of LD downregulates the Nogo-A pathway and increases the oligodendrocyte transcription factor. Therefore, favoring the idea that dopaminergic pathways neuromodulate oligodendrogenesis around the peri-infarct area [17].

Dopamine reuptake plays a vital role in the acute post-stroke setting. Haggman et al. measured the expression of synaptogyrin in aged mice treated with levodopa and compared them to a placebo (saline solution) [18]. Synaptogyrin is a synaptic vesicle protein involved in the reuptake of dopamine. After seven days of treatment, rats were sacrificed and the investigators measured by immunofluorescent stains the dopamine receptors (1 and 2) and the expression of synaptogyrin. As a result, they found that LD treated rats showed more expression of dopamine receptors and a considerable decrease in synaptogyrin receptors, making this a feasible pathway that modulates brain plasticity [18].

## Conclusions

LD alone or in combination with MPD seems to improve motor recovery and mood and promote wakefulness in stroke survivors. Functional, cognitive, and mood recovery in these patients might be explained by LDs' effects of possibly promoting neural plasticity as well as decreasing glucose utilization and increasing motivation. There are several hypotheses as to how LD exerts its effects on motor recovery. Overall, LD seems to be well tolerated among stroke survivors. Research done on animals supports the benefit of administering LD to post-stroke individuals by increasing the oligodendrocyte transcription factor.

Additionally, LD and MPD combined might have a beneficial effect on mood and could be a good alternative to the traditionally used SSRIs. Cognitive improvement with the use of LD was not shown, possibly due to the specific tools used for evaluation. Due to the limited amount of data and clinical trials pertaining to this topic, further research is needed to establish LD as a beneficial drug for post-stroke survivors.
